# Porous Silicon Biosensor for the Detection of Bacteria through Their Lysate †

**DOI:** 10.3390/bios11020027

**Published:** 2021-01-20

**Authors:** Roselien Vercauteren, Audrey Leprince, Jacques Mahillon, Laurent A. Francis

**Affiliations:** 1 Electrical Engineering Department, Institute of Information and Communication Technologies Electronics and Applied Mathematics, UCLouvain, 1348 Louvain-la-Neuve, Belgium; laurent.francis@uclouvain.be; 2 Laboratory of Food and Environmental Microbiology, Earth and Life Institute, UCLouvain, 1348 Louvain-la-Neuve, Belgium; audrey.leprince@uclouvain.be (A.L.); jacques.mahillon@uclouvain.be (J.M.)

**Keywords:** porous silicon membrane, bacterial detection, selective lysis, endolysins, lysostaphin, flow-through

## Abstract

Porous silicon (PSi) has been widely used as a biosensor in recent years due to its large surface area and its optical properties. Most PSi biosensors consist in close-ended porous layers, and, because of the diffusion-limited infiltration of the analyte, they lack sensitivity and speed of response. In order to overcome these shortcomings, PSi membranes (PSiMs) have been fabricated using electrochemical etching and standard microfabrication techniques. In this work, PSiMs have been used for the optical detection of *Bacillus cereus* lysate. Before detection, the bacteria are selectively lysed by PlyB221, an endolysin encoded by the bacteriophage Deep-Blue targeting *B. cereus*. The detection relies on the infiltration of bacterial lysate inside the membrane, which induces a shift of the effective optical thickness. The biosensor was able to detect a *B. cereus* bacterial lysate, with an initial bacteria concentration of 10^5^ colony forming units per mL (CFU/mL), in only 1 h. This proof-of-concept also illustrates the specificity of the lysis before detection. Not only does this detection platform enable the fast detection of bacteria, but the same technique can be extended to other bacteria using selective lysis, as demonstrated by the detection of *Staphylococcus epidermidis*, selectively lysed by lysostaphin.

## 1. Introduction

This paper is the extended version of the proceedings paper presented at the 1st International Electronic Conference on Biosensors, 2–17 November 2020 [1].

A biosensor allows for the fast detection and quantification of a biological analyte, without pre-enrichment steps. It is characterized by two components: a biological element, which is often key to the specificity, and a transducer [2]. The biological element frequently takes the form of a bioreceptor, which is bound to the surface of the transducer. This binding requires several steps of surface modification or functionalization, which can be complex, time-consuming and/or expensive. On top of that, functionalization can shorten the lifespan of the biosensor and often puts heavy requirements on the storage conditions. These bottlenecks can be avoided by adding the biological element into the sample volume instead of binding it to the transducer. Among these biological elements are endolysins: produced by bacteriophages or bacterial cells, they have the capability to specifically digest the cell wall of specific bacterial strains. They have proven to be powerful specificity means for the detection of bacteria [3,4,5].

The transducer can rely on electrical, optical, thermal, or magnetic signals. Optical transducers however outperform most physical transducers as there is no influence of the nature of the sample or of external disturbances on the signal [6]. Optical biosensors can take many forms: waveguides [7], ring resonators [8], refractometers [9,10], surface plasmon resonators [11], optical fibers [12], or even photonic crystals [13]. Porous silicon (PSi) is one widely used optical transducer for biosensors [14]. Its benefits include a large surface area, unique optical properties and a low production cost. While PSi photonic crystal [15], ring resonators [16,17], and photoluminescent sensors [18] have been demonstrated, most PSi-based optical transducers act as interferometers and rely on changes in the effective optical thickness (EOT) of the porous layer as means of detection. The EOT depends on both the effective refractive index and the thickness of the porous layer and can be quantified using reflective interferometric Fourier transform spectroscopy (RIFTS) [19,20]. The RIFTS method consists in shining a halogen light perpendicularly to the sensor surface and measuring its reflection. The reflection takes the form of Fabry-Pérot fringes due to the interferences between the light reflections from the top and the bottom interfaces of the porous film. By applying a Fourier transform to this fringe pattern, it is possible to extract their frequency, which takes the shape of a peak. The position of this peak translates the EOT. When an analyte penetrates the porous matrix, it affects the refractive index of the layer medium and induces a wavelength shift in the interference pattern; this is translated by a shift of the EOT value.

Recent works using the RIFTS as transducing mechanism of PSi-based transducers target the detection of, for instance, bacterial surface proteins [21], heat shock protein 70 [22], or bovine mastitis biomarkers [23,24], but this method has also been applied for the detection of bacteria [25,26,27]. These bacterial detectors however lacked sensitivity, with detection limits unable to go below 10^3^ colony forming units per mL (CFU/mL). Current bacteria detection techniques, such as polymerase chain reaction (PCR) or enzyme-linked immunosorbent assay ELISA, while time-consuming, can easily reach detection limits of 10 CFU/mL [28]. This insufficient sensitivity is attributed to the hindered diffusion of bacteria into the porous matrix. Solutions, such as the electrokinetic transport for the preconcentration of analyte, have been proposed but remain to be tested on bacteria [29]. Recently, porous silicon interferometers have also been combined with gold nanoparticles for localized surface plasmon spectroscopy (LSPS), enhancing the fringe pattern contrast and increasing the sensitivity of the porous layer [30,31].

Another approach to increase the sensitivity of PSi biosensor is the fabrication of PSi membranes (PSiMs). Instead of a close-ended PSi layer over which the analyte must flow, an open-ended PSi membrane allows the analyte to flow through the porous matrix [32]. PSi membranes have been fabricated as early as the 1990s [33], but the interest in PSiMs for sensing applications only arose recently [34,35,36,37,38]. PSiMs can be self-supported, meaning still partly attached to the silicon substrate, or freestanding. Recently, lateral PSiMs have also been fabricated [39,40,41]. For biosensing applications, PSiMs have been found to increase both the response time and amplitude of the sensors [34,35,36,37,38].

In this work, we combine the benefits of selective endolysins and PSiM-based transducers for the fabrication of an innovative biosensor which enables the fast and label-free detection of bacteria through their lysate. The biosensing platform operates in two steps: a first step is the selective lysis of the targeted bacteria by an endolysin in a vial; and secondly, the optical monitoring of the bacterial lysate filtering through a PSiM using the RIFTS method. We demonstrate this concept with a selective optical detection of *Bacillus cereus* lysate in PBS using the recently characterized PlyB221 endolysin, encoded by the Deep-Blue phage targeting *B. cereus* [42]. The specificity is confirmed by replacing the targeted bacteria with *Staphylococcus epidermidis*, for which no optical detection was observed. To illustrate the versatility of our detection platform, the targeted bacteria strain was then switched to *S. epidermidis*, using lysostaphin as selective lytic agent.

## 2. Materials and Methods

### 2.1. Materials

Double-side polished, boron-doped silicon wafers (⟨100⟩, 0.8–0.9 mΩ cm, 380–400 μm) were purchased from Sil’tronix Silicon Technologies (France). Aqueous hydrofluoric acid (HF, 49%) was acquired from Chem-Lab, NV (Belgium), and absolute ethanol was obtained from VWR Chemicals (France). Phosphate buffered saline (PBS, 0.01 M phosphate, pH 7.4) and lysostaphin were purchased from Sigma-Aldrich (USA). 

### 2.2. Fabrication of Porous Silicon Layers

The PSi layer samples were prepared by the electrochemical etch of a heavily doped *p*-type silicon substrate. The etching was carried out in a custom-made Teflon^®^ single bath etch-cell, with a platinum coil as the counter-electrode and a potentiostat/galvanostat (PGSTAT302N from Metrohm Belgium) as the current source. The porosification was performed in HF:ethanol (3:1, in volume) electrolyte. The first step of the anodization consisted in etching a sacrificial layer at 200 mA/cm^2^ for 30 s and removing it with a 2 M solution of KOH until no more reaction was visible. This sacrificial layer removes the transitional layer and obtains a more homogeneous pore size with depth. The sample was then rinsed once in deionized water and twice in 2-propanol before being etched again at 200 mA/cm^2^ for 50 s. The porous samples were thermally oxidized in an oven for 30 min at 350 °C under an oxygen flow (1.2 L/min).

### 2.3. Fabrication and Characterization of Porous Silicon Membranes

**Figure 1:**
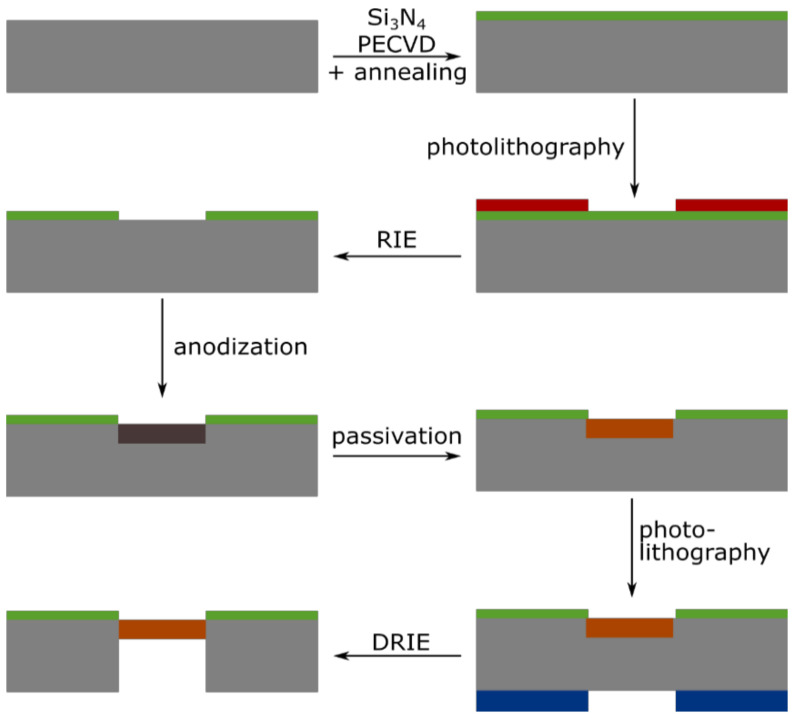
Schematic illustration of the process flow for the fabrication of a porous silicon membrane. Starting from a cleaned 3″ highly doped silicon wafer, the process goes through the following steps: deposition of Si_3_N_4_ layer using Plasma Enhanced Chemical Vapor Deposition (PECVD) and annealing; positive photolithography on the frontside; opening of the nitride layer using Reactive Ion Etching (RIE); formation of the porous silicon layer by anodization; passivation by thermal oxidation; positive photolithography on the backside; and finally opening of the membrane using Deep Reactive Ion Etching (DRIE).

Figure 1 sketches the process flow, which was inspired by the work from Zhao et al. [34]. First, 3-in. highly doped *p*-type silicon wafers were cleaned in a freshly prepared piranha solution (H_2_O_2_:H_2_SO_4_, 2:5), followed by two immersions in continuously flowing deionized (DI) water during 20 min. Afterwards, 500 nm of silicon nitride (Si_3_N_4_) was deposited using Plasma Enhanced Chemical Vapor Deposition (PECVD). To improve the chemical resistance of the nitride layer to HF, the wafers were annealed at 900 °C in ambient air for 3 h. A first i-line optical lithography with positive resist (AZ^®^ MiR^TM^ 701, MicroChemicals GmbH) provided masking for the subsequent Reactive Ion Etching (RIE) of the silicon nitride layer. The patterned nitride layer itself served as a mask during the electrochemical etch of the silicon. The porosification followed the same protocol as explained in Section 2.2, but used three different current densities in order to obtain different porosities: the sensing layer was etched at 200 mA/cm^2^ for 50 s. This was followed by a 1500 s-etch at 50 mA/cm^2^, making a thick optical contrast layer characterized by lower porosity, enabling the reflection of the light required for the RIFTS method; finally, a thick mechanical support layer was etched at 100 mA/cm^2^. The current densities chosen for each layer have been optimized such as to guarantee a good mechanical stability and optical signal, while retaining pores large enough for the flow-through operation. The porous multilayers were passivated by thermal oxidation, for 30 min at 350 °C under an oxygen flow of 1.2 L/min. A second optical lithography was then performed on the backside of the wafers, where a thick positive resist (AZ^®^ 9260, MicroChemicals GmbH) was patterned in alignment with the frontside. The thick resist served as a mask during the final step of the process, the deep reactive ion etching (DRIE) of the backside of the wafer, until the porous silicon was visible and the membranes were open. 

The entire fabrication process was performed in less than a week, and could be repeated several times. Slight variations in the porous structures can happen, which can be linked to the manual positioning of the platinum electrode.

The membranes were characterized using scanning electron microscopy (SEM), both in cross section and in top view. Based on the top views, the pore size distribution could be analyzed using the ImageJ software. The porosity of each layer was determined using the spectroscopic liquid infiltration method (SLIM). In brief, the optical spectrum of a porous layer was recorded both in air and in ethanol. Using the RIFTS method described above, the EOT was calculated. Knowing the refractive indices of air, ethanol and silicon, these data were then fitted using a two-component Bruggeman effective medium approximation in order to obtain an approximation of the open porosity and the layer thickness. The experimental set up used for the SLIM method consisted in a fiber-coupled Ocean Optics JAZ spectrometer and a halogen light source.

Using the EOT measured in both air and ethanol allowed to approximate the theoretical sensitivity of the biosensor [10,31].

### 2.4. PlyB221 Endolysin Expression and Purification

A detailed description of the expression and purification of PlyB221 endolysin can be found elsewhere [42]. The protein concentration was adjusted to 1 mg/mL.

### 2.5. Bacterial Strains, Growth Conditions

*B. cereus ATCC 10987* was used as reference strain and *S. epidermidis* ATCC 35984 as negative control in the PlyB221 endolysin experiments. *S. epidermidis* ATCC 35984 was also used as target when lysostaphin was applied as selective lytic agent. Bacteria were grown overnight (O/N) in Lysogeny Broth (LB) or LB-agar plates at 30 °C for *B. cereus* and in Tryptic Soy Broth (TSB) or Tryptic Soy Agar (TSA) plates at 37 °C for *S. epidermidis*. In brief, 20 mL of LB or TSB were inoculated with 200 µL of each culture and incubated for 3 h at 30 °C (*B. cereus*) or 37 °C (*S. epidermidis*). The cultures were then centrifuged at 10,000× *g* for 5 min at room temperature and the supernatants were resuspended in 20 mL of PBS. This washing step was repeated once over and the optical density (OD_600_) was adjusted to OD_600_ = 0.2 (~10^6^ CFU/mL) for *B. cereus* and OD_600_ = 0.02 (~10^6^ CFU/mL) for *S. epidermidis*. For the determination of the limit of detection, the *B. cereus* suspension was diluted 10 times twice, in order to obtain concentrations of ~10^5^ CFU/mL and ~10^4^ CFU/mL.

### 2.6. Lysate Observation and Characterization

*B. cereus suspension* and lysate were captured on a silicon surface to enable their observation using SEM. *A B. cereus* suspension was prepared as described above and adjusted to the concentrations of ~10^6^ CFU/mL. The lysate was prepared by adding 600 µL of PlyB221 endolysin (1mg/ml) to 5.4 mL of bacterial suspension and by incubating this mixture and 30 °C for 30 min. Silicon dies of dimension 1 cm × 1 cm were place inside a 12 wells plate. The wells were filled with 2 mL of one of three solutions: a control solution consisting of PBS, the *B. cereus* suspension or the *B. cereus* lysate. The 12 wells plate was then incubated at 30 °C for 1 h, after which each die was rinsed 5 times in PBS by removing and adding 1 mL of PBS. After the last PBS wash, 1 mL of solutions remained in each well. A glutaraldehyde solution was added to each well in order to reach a final concentration of 2.5 %. The 12 wells plate was left at room temperature for 1 h, enabling the crosslinking of the bacterial cell walls. The samples were then washed three times with PBS using the same technique as explained before, making sure that the dies were never exposed to air. For the dehydration, the samples were then immersed in DI water solution of increasing ethanol content (25%, 50%, 75%, and finally 99.9%). Each immersion lasted 10 min. The silicon dies were then dried overnight at 58 °C. Directly before SEM observation, the samples were covered with a ~10 nm-thick layer of gold to prevent charging effects. The images were then analyzed with ImageJ to obtain information about the number and size of the bacteria and bacteria lysate.

### 2.7. Experimental Setup and Optical Reflectivity Measurements

PSiM samples were integrated in a custom-built polycarbonate fluidic cell. A fiber-coupled Ocean Optics JAZ spectrometer and a 10-mW halogen light source were used to record reflectivity spectra. Data were recorded every 10 s, with a spectral acquisition time of 1s over a wavelength range of 500–800 nm. Analytes were injected at flow speed of 15 to 20 µL/min using a Fluigent LINEUP™ fluidic set up. The obtained optical data were analyzed using the RIFTS method in order to obtain the effective optical thickness, EOT = *2 nL*, with *n* being the refractive index and *L* the porous layer thickness. The relative change in EOT overtime was computed as a percentage, such that

$$\frac{\Delta EOT}{EOT_{0}}=\frac{EOT_{t}-EOT_{0}}{EOT}\times 100\left[\%\right].$$

The significance of the relative EOT shift was then established using a Student’s t-test with a 5% confidence level, with a negative control test in PBS as reference.

### 2.8. Real-Time Detection of B. cereus in PBS on PSi Layer and PSi Membranes

**Figure 2:**
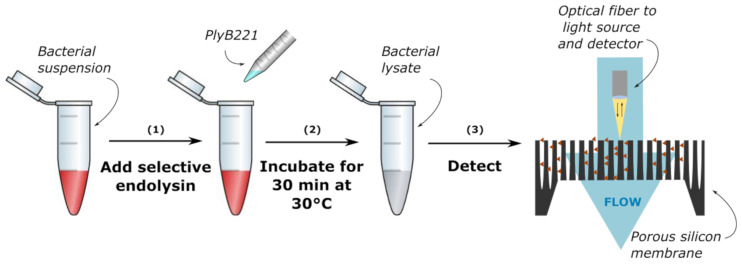
Protocol of bacteria detection through their lysate: (**1**) lysis of the bacteria using a selective endolysin, (**2**) incubation for 30 min and (**3**) optical detection on a porous silicon membrane.

The protocol for bacteria detection on a PSi membrane is illustrated in Figure 2. First 500 µL of purified PlyB221 endolysin were added to 4.5 mL of exponential phase *B. cereus* resuspended in PBS, so as to reach a final protein concentration of 100 µg/mL. The 5 mL final volume was sufficient for at least 4 detections. The suspension was then incubated for 30 min at 30 °C. Before flowing the bacterial lysate, PBS solution was injected at 15 to 20 µL/min for 60 min and reference measurements were performed. *B. cereus* lysate suspensions were injected at the same flow speed. Optical measurements were carried out every 10 s for 60 min. The relative EOT was then extracted from these measurements using the method described above. A control test, with only the PlyB221 endolysin at the same concentration was also performed on both types of sensors, following the same protocol described previously. For all tests, measurements were performed at least 3 times. 

### 2.9. Specificity Testing: Detection of S. epidermidis in PBS with the PlyB221 Endolysin on PSi Membranes

For this experiment, two tests were performed: a negative one using a *S. epidermidis* suspension and a positive control using a complex sample containing both *S. epidermidis* and *B. cereus*. For each test, 4.5 mL of bacterial suspension were incubated for 30 min at 30 °C with the 500 µL of PlyB221 endolysin (1 mg/mL). This final volume was sufficient for at least 4 detections. Reference measurements in PBS were performed for 5 to 15 min. The bacterial suspension was injected at a flow speed of 15 to 20 µL/min and optical measurements were performed every 10 s for 60 min. The relative EOT shift was computed as described previously. 

### 2.10. Versatility of the Platform: Detection of S. epidermidis in PBS with Lysostaphin on PSi Membranes

In this experiment, a different lytic enzyme-bacteria pair was tested. *S. epidermidis* was selected as the targeted strain and lysostaphin was chosen as selective agent. This glycyl-glycine endopeptidase is specific of the pentaglycine bridges present in the cell wall of certain Staphylococci. For the detection experiments, 1 mg of commercialized lysostaphin was diluted in 1 mL of PBS supplemented with 30% of glycerol to reach a concentration of 20 µM. For the lysis, 150 µL of this 20 µM lysostaphin was added to 2.850 mL of bacterial suspension, yielding a final endolysin concentration of 1 µM [4]. The suspension was then incubated at 37 °C for 30 min. Reference measurements in PBS and in lysostaphin suspensions were performed for 1 h each. The bacterial suspension was injected at a flow speed of 15–20 µL/min and optical measurements were performed every minute for 60 min. The relative EOT shift was computed as described previously.

## 3. Results

### 3.1. PSi-Based Biosensor Characterization

**Figure 3:**
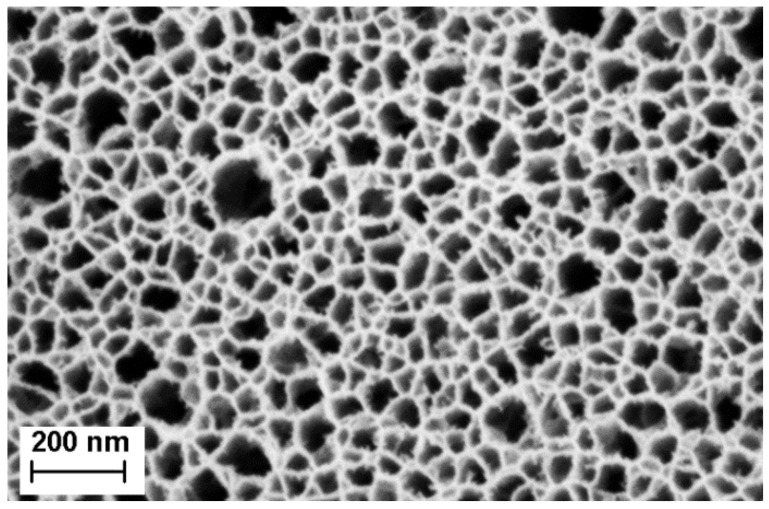
Scanning electron microscopy (SEM) image of the top layer porous silicon membrane. The average pore size is ~41 nm, with a standard deviation of ~20 nm.

**Figure 4:**
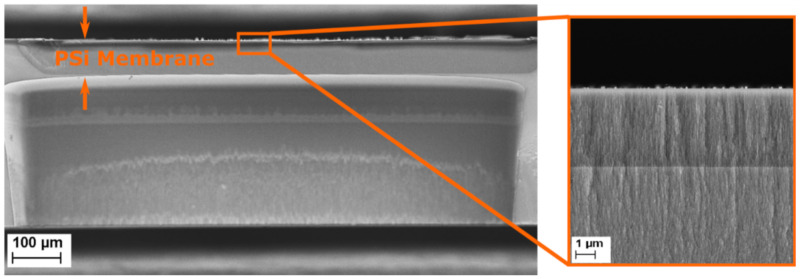
Scanning electron microscopy (SEM) cross-section image of a multilayered porous silicon membrane. The inset shows a close-up of the top of the porous membrane, in which the sensing and the contrast layers can be identified.

The effective optical thickness of Porous Silicon is strongly dependent on its refractive index. When an analyte penetrates the pores, the refractive index of the porous layer increases, therefore inducing a shift in the EOT. In the case of a membrane, to make sure that the analyte remains inside the porous matrix, two approaches are possible: size exclusion or binding to the pore wall. For this project, size exclusion was chosen by accordingly selecting different pore size for each layer of the membrane. The first layer, also called the sensing layer, has an average pore size 41.05 nm, as described in Table 1 and illustrated in Figure 3. Larger pores could be etched, but the resulting layer was too easily damaged, as an increase in pore size induced a decrease of the pore wall thickness. The selected pore size is a trade-off between pore opening and mechanical integrity. The bacterial lysates are composed, among others, of cell wall fragments, DNA and RNA molecules, and cytoplasmic liquid and ribosomes, which are assumed to penetrate the sensing layer. In order to keep the bulkier ones in the top layer, the contrast layer was designed to have a smaller pore size, as indicated in Table 1. These choices in pore size also have two other motivations: (1) they allow the PlyB221 endolysin to flow through the membrane and not be retained in the sensing layer and (2) they prevent the non-lysed bacteria from penetrating the membrane, therefore enabling a selective detection. Figure 4 depicts a PSi membrane, with an up-close view of the transition between the top sensing layer and the contrast layer.

**Figure 5:**
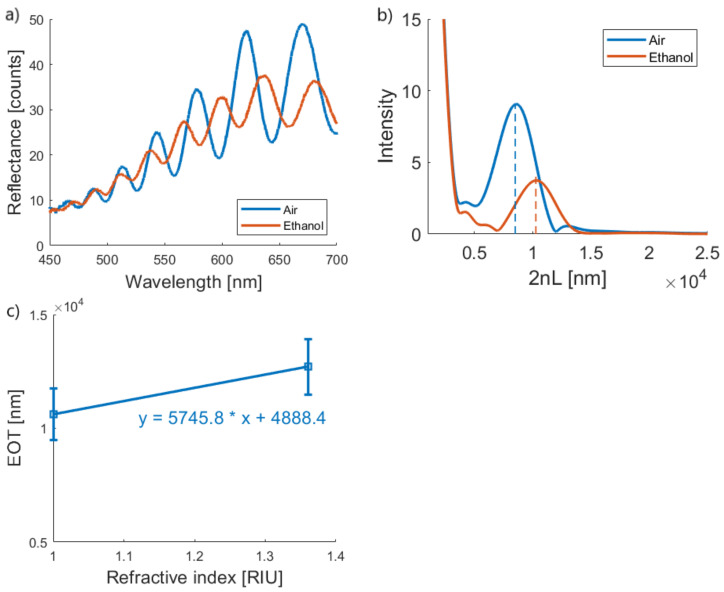
Porous silicon membrane characterization. (**a**) Reflection spectra of a porous silicon membrane in air (blue) and ethanol (orange). (**b**) Representative Fourier transform of the reflectance spectra in panel. (**c**). Calibration curve of the EOT versus the refractive index variation.

The theoretical sensitivity of the porous membrane sensor was also approximated by calculating the EOT in both air and in ethanol, applying a Fourier transform to the Fabry Perot fringes of the optical spectra, as illustrated in Figure 5. By plotting the EOT versus refractive index variation (Figure 5c), an approximation of the sensitivity can be made, which amounts to 5745.8 ± 847.7 nm·RIU^−1^ or, expressed in relative changes of EOT, as 54.4 ± 8.3 %·RIU^−1^.

### 3.2. B. cereus Lysate Observation and Characterization

**Figure 6:**
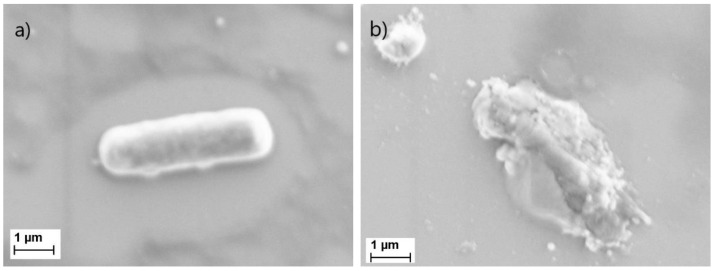
Scanning electron microscopy (SEM) image of (**a**) an intact *B. cereus* bacterium and (**b**) a *B. cereus* lysed bacterium.

Endolysins are encoded and used by bacteriophages at the end of their replication cycle. They degrade the peptidoglycan of the targeted bacteria, creating an opening in the cell wall for the phage. When endolysins are recombinantly produced and added exogenously to bacteria, they lead to cell lysis by breaking down the exposed peptidoglycan layer which make them promising antimicrobial agents [42]. To observe the effect of the PlyB221 endolysin, *B. cereus* was observed using SEM before and after the lysis. Close up images of an intact versus a lysed bacterium are depicted in Figure 6.

To further characterize the bacterial lysate, the average number and size of bacterial clusters were compared before and after lysis, using images of the same magnification. Due to a lack of contrast and sharpness, only qualitative observations could be made. Over the same area of inspection, there are nearly 20 times more clusters of bacterial lysate than of bacteria; the average size of each cluster is also decrease by more than a 10-fold. As illustrated in Figure 6, some larger clusters of bacterial lysate remain, but these are surrounded by much smaller clusters, whose area goes down to the nanometer range (<50 nm). These smaller clusters are assumed to penetrate the first porous layer, but are unable to diffuse to the second porous layer because of a size exclusion effect.

### 3.3. B. cereus Lysate Detection PSi Layers and PSi Membranes

In order to establish the added-value of a flow-through approach to the optical detection with respect to the traditional flow-over approach, the performances of PSi membranes were compared to those measured on PSi layers.

**Figure 7:**
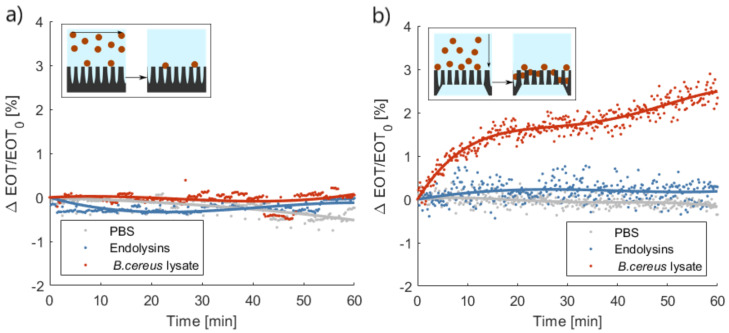
Characteristic relative effective optical thickness (EOT) shift measured on (**a**) a porous silicone (Psi) layer or (**b**) a PSi membrane for 1 h in phosphate buffered saline (PBS), in a PlyB221 endolysin suspension and in a *B. cereus* lysate (*n* ≥ 3). In (**a**), the inset illustrates the flow-over approach; there was no significant shift visible during the detection of either the PlyB221 endolysin or bacterial lysate. In (**b**), the inset illustrates the flow-through approach; there was a significant shift in the relative EOT during the detection of bacterial lysate.

The average relative EOT shift measured on PSi layers, in a flow-over approach, is presented in Figure 7a. No distinction could be made between the bacterial lysate detection and the control tests using either the buffer or the endolysin suspension. Both control tests induced minute decreases of the relative EOT: on average −0.24% and −0.01% after 1 h for PBS and the PlyB221 endolysin, respectively. A small increase of relative EOT was measured in the presence of bacteria lysate, namely 0.05%, but this decrease was not significant when compared to the noise level. This noise level was calculated based on the overall standard deviation of the signal in PBS and was expressed as 3σ = 1.08%.

The performances of PSi membranes for the same three tests in flow-through operation are illustrated in Figure 7b. The buffer control test induced a decrease in relative EOT of −0.32% after 1 h. The control test with only the PlyB221 endolysin gave rise to a 0.28% increase of relative EOT. This increase was however not significant and remained below the noise level, which was calculated in the same manner described previously and was equal to 0.84% in the case of PSi membranes. Upon the penetration of bacterial lysate inside the membrane, a significant increase of relative EOT was measured, exceeding the noise level after 6 min and reaching an average +2.43% after 1 h.

### 3.4. Determination of the Limit of Detection of B. cereus

**Figure 8:**
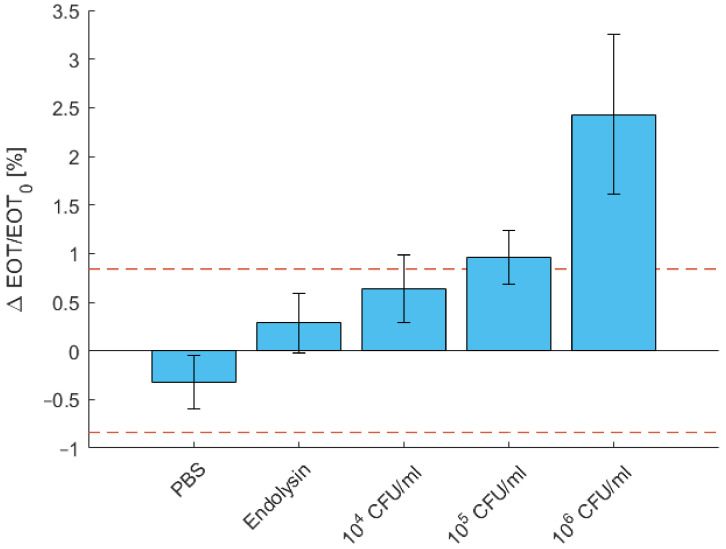
Characteristic relative effective optical thickness (EOT) shift measured on a PSi membrane after 1 h in PBS, in a PlyB221 endolysin suspension and in increasing concentrations of *B. cereus* lysate (*n* ≥ 3). The dashed red line represents the noise level, fixed as 3σ of the signal measured in PBS. The detection limit is 10^5^ CFU/ml of *B. cereus*.

The limit of detection was determined using decreasing concentration of *B. cereus*. Results are displayed in Figure 8. As presented above, the detection of 10^6^ CFU/mL is significant compared to the negative control tests in PBS and in endolysin only. For a concentration of 10^5^ CFU/mL, the relative EOT shift amounted 0.96%, which is just above the noise level but still significantly different to both control tests. For 10^4^ CFU/mL, the relative EOT shift decreased below the noise level to 0.64%. While this difference remains significant with respect to the control test in PBS, it is not with respect to the endolysin control test.

### 3.5. Specificity Testing with S. epidermidis

**Figure 9:**
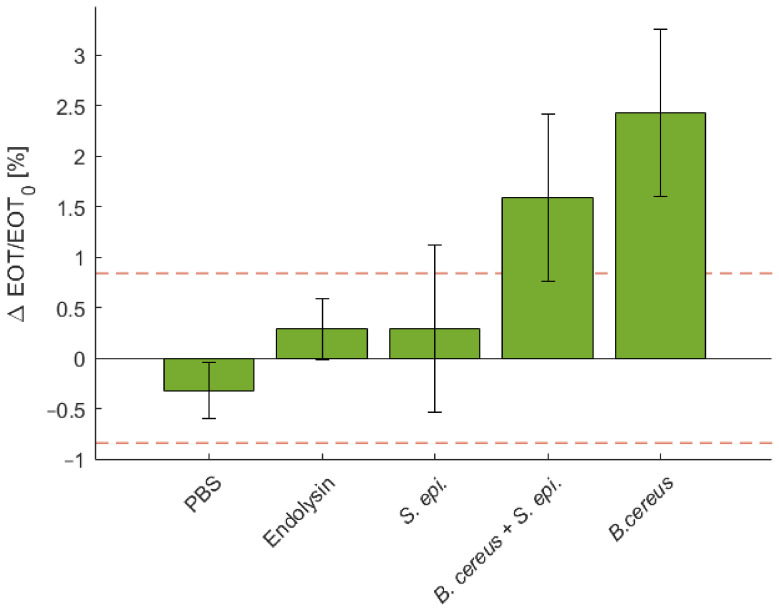
Characteristic relative EOT shift measured on a PSi membrane after 1 h in PBS, in a PlyB221 endolysin suspension, in a *S. epidermidis* suspension, in a mixture of *B. cereus* lysate and *S. epidermidis* and in *B. cereus* lysate only (*n* ≥ 3). The dashed red line represents the noise level, fixed as 3σ of the signal measured in PBS. Only the detection of mixture of *B. cereus* lysate and *S. epidermidis* induced a significant shift.

To illustrate the specificity of the sensing platform, a negative control test was performed using a *S. epidermidis* and a positive control test was carried out using a mixture of *B. cereus* and *S. epidermidis*. The relative EOT shifts observed for both tests are depicted in Figure 9, where it is compared to controls in PBS and with the PlyB221 endolysin only, as well as to the detection of *B. cereus* lysate only. The detection using *S. epidermidis* induced no significant shift in relative EOT. After 1 h, the relative EOT was increased by 0.29%, which is comparable to the control test with only the PlyB221 endolysin. The positive control test showed a significant relative EOT increase of 1.59%.

### 3.6. Versatility of the Platform: Detection of S. epidermidis in PBS with Lysostaphin

**Figure 10:**
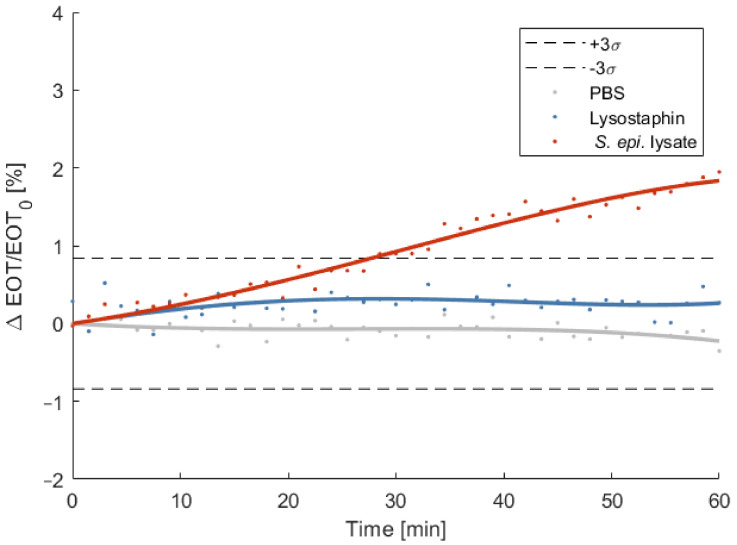
Relative effective optical thickness (EOT) shift measured on a PSi membrane for 1 h in PBS (grey), in a lysostaphin suspension (blue) and in a *S. epidermidis* lysate (red). The bacterial lysate is detected after 30 min.

To illustrate that the biosensor can be used for the detection of other bacteria/lytic enzyme pairs, the same detection protocol was repeated using *S. epidermidis* as targeted strain and lysostaphin as selective lytic agent. The relative EOT shift was measured overtime and compared to control tests in PBS and lysostaphin only. As depicted on Figure 10, the relative EOT shift induced after 1 h by the penetration of lysostaphin into the porous membrane is comparable to the one measure for the PlyB221 endolysin and amounts 0.26%. The penetration of *S. epidermidis* lysate causes a significant shift in relative EOT after 30 min, reaching a value of 1.83% after 1 h.

## 4. Discussion

Porous silicon membranes are promising biosensors: with their flow-through operation, they overcome the lack of sensitivity of flow-over PSi-based biosensors and are characterized by short response times [34,35,36,37,38]. Moreover, they do not require any stratagems to transport and concentrate the analyte on the transducer. In this work, we demonstrate once again the potential of these detection platforms and focus on their use for the detection of bacteria. PSiMs were fabricated using standard microfabrication techniques and electrochemical etching. The full fabrication process took less than a week, and dozens of samples could be produced in one attempt. No functionalization was applied to the sensors, as the specificity was based on the use of endolysins. Endolysins are phage-encoded enzymes that induce bacterial lysis for certain targeted strains. In this work, the PlyB221 endolysin was used, targeting *B. cereus*, whose efficiency is demonstrated in [42]. The physical effect of PlyB221 endolysin on the bacteria was observed using SEM. While large clusters of bacterial lysate remained, they were surrounded by nanoscale-sized clusters. It is assumed that these small clusters are able to penetrate the porous membrane and enable a detection.

Combining PSiMs and the use of endolysins produced an innovative biosensing platform that was able to detect *B. cereus* lysate, with an initial concentration of 10^6^ CFU/mL, in less than 10 min. In flow-over PSi-based biosensors, similar concentrations could not be detected. Two negative control tests were carried out: one in PBS and one with the PlyB221 endolysin only. The PBS control induced a slight decrease of relative EOT. This can be explained by the slow oxidation and dissolution that PSi undergoes in aqueous media [43,44]. This effect can be minimized by chemically modifying the pore surface using either hydrosilylation [45], thermal hydrocarbonization [46] or atomic layer deposition (ALD) of oxides [47]. Adequate passivation may also help reducing the noise level of the optical sensing, as demonstrated by Rasson et al. with the use of ALD [47]. The second control test, which consisted in flowing a PlyB221 endolysin suspension through the sensor for 1 h, resulted in a slight increase of the relative EOT. While it was expected that the endolysins pass through the membrane, this increase indicates that they were partly retained. We believe two hypotheses might explain this effect: (1) a minor size exclusion effect, which is understandable since there is a large pore size distribution visible in Figure 3 and (2) the binding of proteins to the pore walls. 

In order to determine the detection limit, the initial concentration of bacteria was reduced. The sensor was able to detect concentrations as low as 10^5^ CFU/mL after 1 h. While concentration of 10^4^ CFU/mL induce a significant increase in signal compared to the PBS control test, this increase was however not significant with respect to the endolysin control test. Once again, adequate passivation may help to reduce the deviation between measurements and enable the lowering of the detection limit to 10^4^ CFU/mL or lower.

While the specificity of the PlyB221 endolysin has already been demonstrated [42], the specificity of the sensor was still exemplified by adding *S. epidermidis* to the PlyB221 endolysin suspension. The observed shift in relative EOT is similar to the one observed with the PlyB221 endolysin only. This confirms that bacteria, when not lysed, are not able to penetrate the membrane, thus demonstrating the specificity of the sensing platform. A second test was performed adding both *B. cereus* and *S. epidermidis* to the endolysins and the results showed a significant increase in relative EOT, but lower than the one measured for *B. cereus* lysate only. We believe this might be explained by the accumulation of *S. epidermidis* on top of the sensor, blocking part of the pore and preventing the penetration of *B. cereus* lysate. This accumulation of intact bacteria may also explain the larger deviation between measurements when in the presence of *S. epidermidis*: part of the light is scattered by the bacteria, therefore decreasing the optical signal and adding deviations during the fit of the EOT. A solution to minimise this effect is the addition of gold nanoparticles to the porous matrix, enhancing the optical signal by increasing surface reflectivity and reducing EOT fitting deviations [30].

While most PSiM-based sensors rely on functionalization to capture the analyte [34,37,38], the biosensor presented in this work bases the capture on a size exclusion effect. This novelty has several benefits: the lack of functionalization considerably shortens the production time of the sensors and puts no requirements on the storage and detection conditions. This choice of design was made with future industrial specifications in mind: without the constraints of functionalization, a silicon-based device such as the one presented, which is compatible with all standard fabrication techniques used in microelectronic cleanrooms today, could be easily mass produced and packaged, for a very low cost. The lack of functionalization also enables the use of sensors from the same batch for the detection of different strains of bacteria, as long as a selective endolysin is available. This versatility was illustrated by the detection of *S. epidermidis* using lysostaphin as selective lytic agent. 

Besides specificity, the performance of our optical biosensor can be discussed in other terms: response time, sensitivity and limit of detection [10]. The response time for this study is comparable to functionalized PSiMs [34,37,38] and since no rinsing is required to remove unbound species, the total detection time (which includes both lysis and optical monitoring) can be reduced to less than 2 h. Another major advantage is the reduced volume of analyte that is needed: only 1 mL is required for the detection. The approximated theoretical sensitivity of 5745.8 ± 847.7 nm·RIU^−1^ is high, but due to the noisy signal, the theoretical limit of detection is also quite high; this value was extrapolated as the RIU value at which the relative EOT shift is equal to the noise level 3σ and amounts to 1.5 × 10^−2^ RIU [30]. In terms of bacterial concentration, a limit of detection of 10^5^ CFU/mL remains high compared to other PSi bacteria sensors [25,26,48,49], yet there is much room for improvement. Further studies should therefore aim to lower the detection limit to competitive levels (<10^3^ CFU/mL) by improving the stability of the sensor and reducing the noise level of the optical signal. Once an optimized prototype is available, real samples may be analysed. A complex microfluidic integration may also enable the optical monitoring of several membranes in parallel, adding the possibility for a blank control test (with the analysed sample only) to exclude false positives, as well as for the simultaneous screening of several lytic agents.

## 5. Conclusions

In conclusion, we demonstrated the proof-of-concept of an innovative biosensor for the detection of bacteria through their lysate, by combining the use of both optically monitored porous silicon membranes and selective enzymatic lytic agents. We showed promising results in terms of sensitivity, specificity and speed of response by confirming the targeted detection of 10^5^ CFU/ml of *B. cereus* lysate in only one hour. Furthermore, we illustrated the versatility of the detection platform by detecting selectively lysed *S. epidermidis*. These results, added to the easy fabrication and low design cost, pave the way for the development of a widespread multi-strain bacteria sensor.

## Figures and Tables

**Table 1:** Pore diameter, thickness and porosity measured for each of the three layers of the porous silicone membrane (PSiM).

Layer	Current Density [mA/cm^2^]	Time [s]	Pore diameter [nm]	Thickness [µm]	Porosity [%]
Sensing layer	200	50	41.05 ± 20.4	4.09 ± 0.7	75.4
Contrast layer	50	1500	14.6 ± 7.8	22.8 ± 6.8	48.5
Support layer	100	2000	25.5 ± 10.4	- *	- *

* The thickness and porosity could not be accurately measured, as part of the layer was etched away during the DRIE step.

## Data Availability

The data presented in this study are openly available in Zenodo at 10.5281/zenodo.4446911, reference number data.xlsx.
